# Kojic Acid Showed Consistent Inhibitory Activity on Tyrosinase from Mushroom and in Cultured B16F10 Cells Compared with Arbutins

**DOI:** 10.3390/antiox11030502

**Published:** 2022-03-04

**Authors:** Wei Wang, Ying Gao, Weiwei Wang, Jianyong Zhang, Junfeng Yin, Ting Le, Jinjin Xue, Ulrich H. Engelhardt, Heyuan Jiang

**Affiliations:** 1 Key Laboratory of Tea Biology and Resources Utilization, Ministry of Agriculture, Tea Research Institute, Chinese Academy of Agricultural Sciences, 9 Meiling South Road, Xihu District, Hangzhou 310008, China; wangwei@tricaas.com (W.W.); yinggao@tricaas.com (Y.G.); wangwei11211@tricaas.com (W.W.); zjy5128@tricaas.com (J.Z.); yinjf@tricaas.com (J.Y.); letin@tricaas.com (T.L.); xuejinjin@tricaas.com (J.X.); 2 Graduate School of Chinese Academy of Agricultural Sciences, 12 Zhongguancun South Street, Haidian District, Beijing 100081, China; 3 Institute of Food Chemistry, Technischen Universität Braunschweig, Schleinitzstr. 20, 38106 Braunschweig, Germany; u.engelhardt@tu-bs.de

**Keywords:** tyrosinases, kojic acid, α-arbutin, β-arbutin, deoxyarbutin, monophenolase activity, diphenolase activity, inhibition type, melanin content, cell viability

## Abstract

Kojic acid, β-arbutin, α-arbutin, and deoxyarbutin have been reported as tyrosinase inhibitors in many articles, but some contradictions exist in their differing results. In order to provide some explanations for these contradictions and to find the most suitable compound as a positive control for screening potential tyrosinase inhibitors, the activity and inhibition type of the aforementioned compounds on monophenolase and diphenolase of mushroom tyrosinase (MTYR) were studied. Their effects on B16F10 cells melanin content, tyrosinase (BTYR) activity, and cell viability were also exposed. Results indicated that α-arbutin competitively inhibited monophenolase activity, whereas they uncompetitively activated diphenolase activity of MTYR. β-arbutin noncompetitively and competitively inhibited monophenolase activity at high molarity (4000 µM) and moderate molarity (250–1000 µM) respectively, whereas it activated the diphenolase activity of MTYR. Deoxyarbutin competitively inhibited diphenolase activity, but could not inhibit monophenolase activity and only extended the lag time. Kojic acid competitively inhibited monophenolase activity and competitive–noncompetitive mixed-type inhibited diphenolase activity of MTYR. In a cellular experiment, deoxyarbutin effectively inhibited BTYR activity and reduced melanin content, but it also potently decreased cell viability. α-arbutin and β-arbutin dose-dependently inhibited BTYR activity, reduced melanin content, and increased cell viability. Kojic acid did not affect cell viability at 43.8–700 µM, but inhibited BTYR activity and reduced melanin content in a dose-dependent manner. Therefore, kojic acid was considered as the most suitable positive control among these four compounds, because it could inhibit both monophenolase and diphenolase activity of MTYR and reduce intercellular melanin content by inhibiting BTYR activity without cytotoxicity. Some explanations for the contradictions in the reported articles were provided.

## 1. Introduction

Excessive melanin synthesis involves many negative aspects of life, such as hyperpigmentation in the epidermis, affecting aesthetics and greatly increasing the risk of malignant melanoma [1], and enzymatic browning of fruits and vegetables, resulting in the undesirable reduction in nutrition and consumer acceptance [2,3]. Tyrosinase (EC 1.14.18.1) is a key enzyme for the biosynthesis of melanin and catalyzes two types of reaction: (a) monophenolase activity for the conversion of L-tyrosine into 3,4-dihydroxyphenylalanine (L-DOPA), and (b) diphenolase activity for the oxidizing L-DOPA to o-dopaquinone. Inhibiting the activity of tyrosinase is an effective and important way to avoid melanin synthesis.

Positive controls can be used to measure the inhibitory strength of a potentially effective inhibitor. It is not easy to be influenced by the varied assay conditions, such as different substrate concentrations, incubation time, and different sources of tyrosinase [4]. Kojic acid and β-arbutin (also called arbutin) are well-known depigmenting agents and tyrosinase inhibitors [5,6,7]. They are commonly used as positive controls for screening emerging components or extracts that effectively inhibit melanin synthesis [5,8,9]. Wang et al. screened effective antimelanogenic compounds from tea catechins and their dimeric oxidation products partly by comparing their effect with kojic acid [10]. Except for β-arbutin, many studies also pay attention to α-arbutin (synthetic counterpart of β-arbutin) and deoxyarbutin (derivative of β-arbutin), both of which could inhibit melanogenesis or tyrosinase activity in human or animal skin [11,12], cell system [13], and cell free system [14]. α-arbutin is obtained principally by enzymatic synthesis from hydroquinone or β-arbutin [6,15,16]. The structure difference of β-arbutin and α-arbutin lie in the β- or α-anomer of D-glucose bounded to hydroquinone [17,18]. After all hydroxyls on glycoside side chain are removed, β-arbutin is converted to deoxyarbutin. This procedure results in an increased liposolubility of deoxyarbutin compared with that of β-arbutin. Deoxyarbutin was an effective inhibitor of mushroom tyrosinase (MTYR) with more potency than hydroquinone and β-arbutin [11,19].

However, there were some contradictions among the research results that have been reported in recent years. Firstly, a number of papers confirmed that β-arbutin could reduce cellular melanin content by decreasing intracellular tyrosinase activity [20] and inhibit the activity of MTYR [21]. However, Nakajima et al. reported that the pigmentation in cultured normal human melanocytes became darker (2–8 mM), whereas the viability (2–8 mM) and the tyrosinase activity (0.5–4 mM) of the cells decreased in a dose-dependent manner after being treated with β-arbutin [22]. Secondly, Funayama et al. reported that α-arbutin does not inhibit diphenolase activity of MTYR (unlike β-arbutin with IC_50_ of 8.4 mM), but it is 10 times more potent than β-arbutin as an inhibitor of tyrosinase diphenolase from B16 mouse melanoma cells [23]. That is, α-arbutin and β-arbutin show different activities on tyrosinase of different species. Thirdly, compounds showed different inhibition types in different experiments. Funayama et al. speculated that the inhibitory type of α-arbutin against the tyrosinase from B16 cells was a mixed-type inhibition [23]. However, the inhibitory type of α-arbutin against human tyrosinase (human malignant melanoma cells, HMV-II) was indicated to be competitive in another study [13].

Trying to resolve the above contradictions in the reported articles, the activities of kojic acid, α-arbutin, β-arbutin, and deoxyarbutin on tyrosinase from mushroom and in B16F10 mouse melanoma cells were studied using L-tyrosine or L-DOPA as the substrates. The characteristics of each compound were comprehensively compared in order to: (1) determine which compound is the most appropriate positive control for screening emerging components or extracts that effectively inhibit tyrosinase activity in cell free system and cell system; (2) expose activity difference and inhibition type of α-arbutin on monophenolase and diphenolase, as kojic acid, β-arbutin, or deoxyarbutin; (3) compare diphenolase activity difference between cell free system and cell system of α-arbutin, as kojic acid, β-arbutin, or deoxyarbutin; (4) compare tyrosinase inhibitory activity of kojic acid, α-arbutin, β-arbutin, and deoxyarbutin in a cell-free system or cell system. This study could screen appropriate positive controls for the detection of tyrosinase inhibitory activity in a cell-free system or cell system. It also exposed activity difference of these control compounds in different tyrosinase activities (monophenolase and diphenolase) and different systems (cell or cell-free).

## 2. Materials and Methods

### 2.1. Reagents

**Figure 1:**
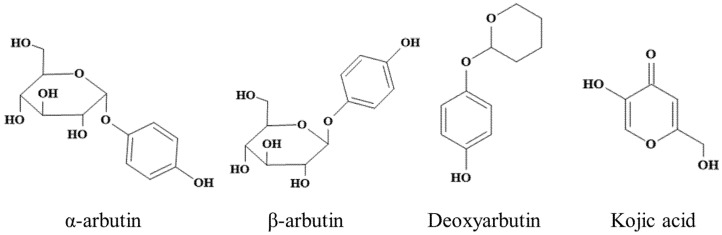
Chemical structure of α-arbutin, β-arbutin, deoxyarbutin, and kojic acid.

Kojic acid (≥99%) and L-DOPA were purchased from Macklin (Shanghai, China). L-(-)-tyrosine were purchased from TCI (Shanghai) Development Co., Ltd. Commercial MTYR (S10171-100KU-1), α-arbutin (≥98%), β-arbutin (≥99%), and deoxyarbutin (≥99%) was purchased from Shanghai Yuanye Bio-Technology Co., Ltd. (Shanghai, China). Chemical structures of α-arbutin, β-arbutin, deoxyarbutin, and kojic acid were shown in Figure 1.

### 2.2. MTYR Activity Assay

The activity of MTYR was determined by measuring the rate of dopachrome formation at 475 nm with Synergy H1 microplate reader (BioTek Instruments, Inc., Winooski, VT, USA) according to the literature procedure with minor modification [7,24]. L-tyrosine and L-DOPA were used as the substrates to test the monophenolase activity and diphenolase activity, respectively. Freshly prepared tyrosinase and substrate solutions were used in this experiment. For air-saturated solutions, a temperature of 25 °C and pH of 6.8 was maintained throughout the study [7]. All assays were performed in triplicate.

#### 2.2.1. Determination of Monophenolase Activity

In flat-bottom 96-well plates, the reaction media was 200 µL. MTYR (40 µL, 300 U/mL) in phosphate buffer saline (20 mM, pH 6.8) was incubated with the corresponding compound (40 µL) at 25 °C for 10 min. Then, L-tyrosine (40 µL, 2 mM) was added to each well as a substrate. Once the substrate was added in reaction system, the formation of dopachrome was immediately measured at 475 nm for 30 min at 1 min intervals. The concentrations of kojic acid were 7.5, 15, 30, 60, and 120 µM. The concentrations of α-arbutin were 480, 960, 2000, 4000, and 8000 µM. The concentrations of β-arbutin were 240, 480, 960, 2000, and 4000 µM. The concentrations of deoxyarbutin were 3.75, 7.5, 15, 30, and 60 µM. These concentrations were used to determine the progress curves and inhibition rates of monophenolase. The inhibition percentage of monophenolase activity was calculated according to the following formula.

Δsample = sample − sample control, Δcontrol = negative control − blank control(1)

k_Δsample_ = (Δsample_t1_ − Δsample_t0_)/(t1 − t0), k_Δcontrol_ = (Δcontrol_t1_ − Δcontrol_t0_)/(t1 − t0) (2)

(3) $$\mathrm{Inhibition}\;\left(\%\right)=\left(1-\frac{k_{∆\mathrm{sample}}}{k_{∆\mathrm{control}}}\right)\times 100$$

In the formula, ‘negative control’ is the treatment without sample; ‘blank control’ is the treatment without sample and substrate; ‘sample’ is the treatment including all solutions; and ‘sample control’ is the treatment without substrate but including sample. If the inhibition rate was negative, it meant the tyrosinase was activated. The activation rate was the absolute value of inhibition rate.

#### 2.2.2. Determination of Diphenolase Activity

In this method, the concentrates of MTYR and L-DOPA were 30 U/mL and 8 mM, respectively. The concentrations of kojic acid to determine inhibition rate of diphenolase were 30, 60, 120, 240, 480, and 960 µM. The concentrations of α-arbutin, β-arbutin, and deoxyarbutin to determine inhibition rate of diphenolase were 30, 60, 120, 240, 480, 960, 2000, 4000, and 8000 µM. Additionally, three concentrations of every compound were selected to present progress curves. Once the substrate was added in reaction system, the formation of dopachrome was immediately measured at 475 nm for 25 min at 1 min intervals. The other experimental steps and calculated formulae are consistent with those of Section 2.2.1.

### 2.3. Kinetic Analysis of MTYR

The experimental method was the same as the MTYR activity assay (Section 2.2), except for the substrate concentration and compound concentration.

The concentration of enzyme was fixed, while the concentration of substrate was changed. The concentrations of L-tyrosine were 0.125, 0.25, 0.5, 1, and 2 mM. The concentrations of L-DOPA were 0.0625, 0.25, 1, 4, and 8 mM. When determining inhibition type of monophenolase, the concentration of kojic acid were 7.5, 30, and 120 µM. The concentrations of α-arbutin were 500 and 2000 µM. The concentrations of β-arbutin were 250, 1000, and 4000 µM. The concentrations of deoxyarbutin were 3.75, 15, and 60 µM. When determining inhibition type of diphenolase, the concentration of kojic acid were 7.5, 30, and 120 µM. The concentrations of α-arbutin and β-arbutin were 500, 2000, and 8000 µM. The concentrations of deoxyarbutin were 7.5, 30, and 120 µM. Once the substrate was added to the reaction system, the formation of dopachrome was immediately detected at 475 nm once every minute for 30 min. Steady state rate (*V*_0_) was defined as the slope of the linear range of progress curves, also called the dopachrome accumulation curve [7]. The *K*_m_ (substrate concentration that yield a half-maximal velocity) and *V*_max_ (maximum velocity) of tyrosinase were determined by Michaelis–Menten equation. Lineweaver–Burk plot was used to display the data.

### 2.4. Cellular Assays

Measurement of B16F10 cellular tyrosinase (BTYR) activity, melanin content, and cell viability were the same as our previous study [10]. Blank group (control 1) was the treatment containing cells and medium. Control group (control 2) was the treatment containing cells, medium, and α-MSH. Compound groups were the treatments containing cells, medium, α-MSH, and related compounds. Experiments were performed at least in triplicate.

#### 2.4.1. Cell Culture

B16F10 mouse malignant melanoma cell line from Stem Cell Bank (Chinese Academy of Sciences, Shanghai, China) were cultured in DMEM supplemented with 10% fetal bovine serum (FBS; Gibco, Grand Island, NV, USA) and antibiotics (100 U/mL penicillin and 100μg/mL streptomycin) in a humidified atmosphere of 5% CO_2_ at 37 °C. B16F10 cells at the exponential phase were adjusted for cell density and used in the following assay [10].

#### 2.4.2. Measurement of BTYR Activity

The BTYR activity was measured as the L-DOPA oxidase activity. B16F10 cells were seeded and incubated for 24 h in 24-well cell culture plates. Then, they were treated with 50 µL of 1 µM α-MSH and different molarities of compounds for another 48 h. After being washed twice with cold D-PBS and lysed with a lysis buffer (P0013J) containing 1% Triton X-100 for 30 min at −20 °C, the lysates were centrifuged at 12,000 rpm for 10 min at 4 °C. The supernatant was used as the BTYR solution. The reaction mixture, containing 50 μL of cell lysate supernatant and 50 μL of 5 mM L-DOPA, was incubated at 37 °C for 1 h and the level of dopachrome formation was measured spectrophotometrically at 475 nm. Lysis buffer was used as the control. The BTYR activity was calculated as a percentage of the control.

#### 2.4.3. Measurement of Cellular Melanin Content

Briefly, B16F10 cells were treated with compounds as in Section 2.4.2. After being washed with PBS and dissolved in 1 M NaOH containing 10% DMSO for 1 h at 80 °C, the cell lysates were centrifuged at 12,000 rpm for 10 min. 150 μL of supernatant was transferred to 96-well plates and the absorbance was measured at 405 nm. The melanin content is expressed as a percentage of the control.

#### 2.4.4. Measurement of Cell Viability

The cytotoxicity of kojic acid, α-arbutin, β-arbutin, and deoxyarbutin against B16F10 were evaluated by using the CCK-8 assay. Cells were seeded into 96-well plates at a density of 1 × 10^4^ cells/well and incubated for 24 h. Then, the cells were treated with different concentrations of compounds for 48 h. After rinsing once with D-PBS, 100 uL DMEM containing 10% CCK-8 was added and incubated for another 1 h. The absorbance values of the solution at 450 nm were measured. The cell viability is expressed as a percentage of the control.

### 2.5. Statistical Analysis

The results are presented as mean ± standard deviation (SD). Comparisons between the two groups were performed with Student’s *t*-test, and one-way analysis of variance (ANOVA) with Duncan’s post hoc test were performed to measure the significant differences among multiple comparisons between compound effects. * *p* < 0.05 and ** *p* < 0.01 were used to consider statistical significance. Michaelis–Menten equation and Lineweaver–Burk plot were analyzed and drawn using GraphPad Prism (Version 9.00, GraphPad Software Inc., San Diego, CA, USA). Chemical structures were drawn by KingDraw (Version 1.1.1, KingDraw, Shangdong, China).

## 3. Results

### 3.1. Inhibitory Effect of Kojic Acid, α-Arbutin, β-Arbutin, and Deoxyarbutin on Monophenolase Activity of MTYR

**Figure 2:**
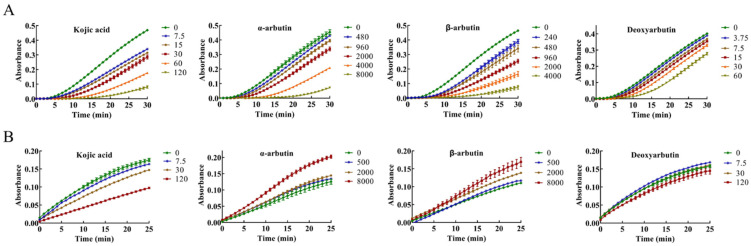
Progress curves of kojic acid, α-arbutin, β-arbutin, and deoxyarbutin on monophenolase and diphenolase of mushroom tyrosinase (MTYR). (**A**) Progress curves of kojic acid (7.5–120 µM), α-arbutin (480–8000 µM), β-arbutin (240–4000 µM), and deoxyarbutin (3.75–60 µM) on monophenolase of MTYR. (**B**) Progress curves of kojic acid (7.5, 30, and 120 µM), α-arbutin (500, 2000, and 8000 µM), β-arbutin (500, 2000, and 8000 µM), and deoxyarbutin (7.5, 30, and 120 µM) on diphenolase of MTYR.

The inhibitory effect of kojic acid, α-arbutin, β-arbutin, and deoxyarbutin on monophenolase of MTYR was investigated, and the reaction process curve of catalyzing L-tyrosine into dopachrome was shown in Figure 2A. The lag time of kojic acid for monophenolase activity was extended in a molarity-dependent manner, so too with α-arbutin, β-arbutin, and deoxyarbutin. After the lag period, the system reached a steady state rate that the absorbance increased linearly with time.

**Figure 3:**
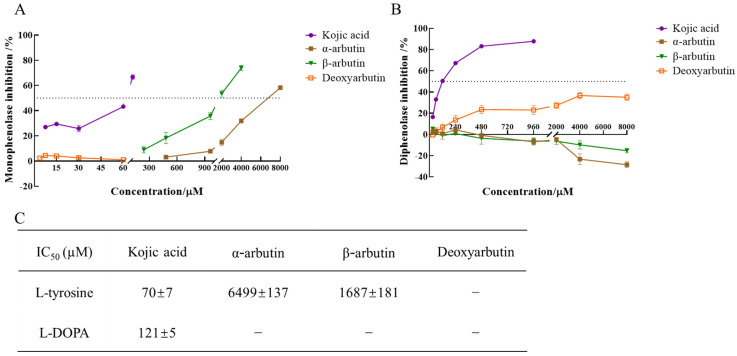
IC_50_ value of kojic acid, α-arbutin, β-arbutin, and deoxyarbutin on monophenolase and diphenolase of MTYR. (**A**) Inhibition rate of monophenolase by compounds; (**B**) Inhibition rate of diphenolase by compounds; (**C**) IC_50_ value of compounds on monophenolase and diphenolase. If the inhibition rate was negative, it meant the tyrosinase was activated. The activation rate was the absolute value of inhibition rate.

The enzyme activity, which was reflected as the slope of the linear range of the kinetic curve, was reduced with the increasing molarity of kojic acid, α-arbutin, and β-arbutin in the reaction system. However, deoxyarbutin did not influence the slope of the linear range of the kinetic curve, so deoxyarbutin could not inhibit monophenolase activity of MTYR. As shown in Figure 3A,C, kojic acid showed the most potent tyrosinase inhibitory activity with IC_50_ value of 70 ± 7 μM compared to arbutins. The inhibitory effect of α-arbutin (IC_50_ value of 6499 ± 137 μM) on monophenolase of MTYR was weaker than β-arbutin (IC_50_ value of 1687 ± 181 μM). Therefore, the results illustrated that the effect of kojic acid, β-arbutin, and α-arbutin on monophenolase of MTYR was both to extend the lag time and to reduce the enzyme activity in the steady state, while deoxyarbutin only extend the lag time.

### 3.2. Kinetic Analysis on Monophenolase

In this experiment, constant concentration of enzyme and changed concentration of L-tyrosine were used to measure the effects of different concentrations of compounds on monophenolase activity.

**Figure 4:**
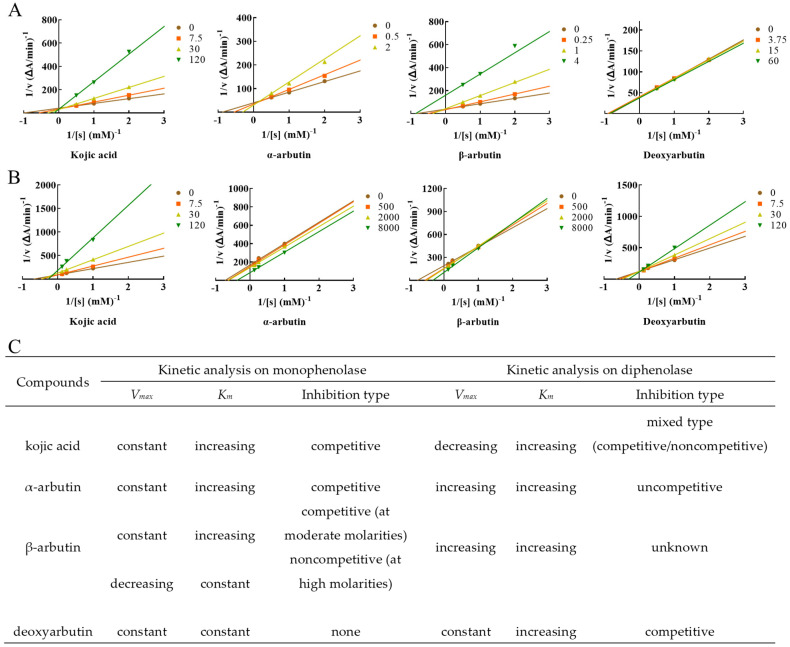
Determination of the inhibition type of kojic acid, α-arbutin, β-arbutin, and deoxyarbutin on monophenolase and diphenolase from MTYR. (**A**) The Lineweaver–Burk plot for kojic acid, α-arbutin, β-arbutin, and deoxyarbutin on monophenolase; (**B**) The Lineweaver–Burk plot for kojic acid, α-arbutin, β-arbutin, and deoxyarbutin on diphenolase; (**C**) Kinetic parameters and inhibition type of kojic acid, α-arbutin, β-arbutin, and deoxyarbutin on monophenolase and diphenolase. Changes of *V*_max_ and *K*_m_ with the increasing compound molarity were shown.

Lineweaver–Burk plot was used to display the inhibition type. The plots of 1/V versus 1/[S] gave a series of lines with different slopes and intersecting at the *y*-axis (Figure 4). Enzyme kinetic analysis indicated that kojic acid was a competitive inhibitor (intersection on *y* axis), *V*_max_ remained constant and *K*_m_ increased with increasing concentrations of kojic acid, so too with α-arbutin. When at moderate molarity (250–1000 µM), *K*_m_ increased, but *V*_max_ remained constant with increasing concentration of β-arbutin, which was competitive inhibitor (intersection on *y*-axis). When at high molarity (4000 µM), *K*_m_ remained constant, but *V*_max_ decreased of β-arbutin compared to no β-arbutin group (intersection on *x* axis), which was noncompetitive inhibitor. In addition, *K*_m_ and *V*_max_ remained constant with increasing concentration of deoxyarbutin, this indicated deoxyarbutin did not influence monophenolase activity. This result of deoxyarbutin was consistent with that of 3.1 (Figure 3A,C).

### 3.3. Inhibitory Effect of Kojic Acid, α-Arbutin, β-Arbutin, and Deoxyarbutin on Diphenolase Activity of MTYR

The diphenolase activity of MTYR was investigated using L-DOPA as a substrate. There was no lag time for kojic acid, α-arbutin, β-arbutin, and deoxyarbutin on diphenolase activity (Figure 2B). As shown in Figure 3B,C, kojic acid showed potent tyrosinase inhibitory activity with IC_50_ values of 121 ± 5 µM. Deoxyarbutin (30–8000 µM) inhibited tyrosinase activity in a dose-dependent manner, but did not reach half maximal inhibitory concentration (IC_50_) in experimental molarity. Conversely, α-arbutin and β-arbutin prompted tyrosinase activity in a dose-dependent manner, which was more obvious at higher molarities (4000–8000 µM). Tyrosinase promoting activity of α-arbutin at 4000–8000 µM was stronger than β-arbutin.

### 3.4. Kinetic Analysis on Diphenolase

L-DOPA was used as the substrate to measure inhibition type of these compounds on diphenolase of MTYR. Enzyme kinetic analysis indicated that kojic acid was a competitive–noncompetitive mixed-type inhibitor (intersection on second quadrant), *V*_max_ decreasing and *K*_m_ increasing with increasing concentrations of kojic acid, which interacted with both free enzyme and enzyme–substrate complex. On the other hand, results indicated that *K*_m_ and *V*_max_ values increased with increasing concentrations of α-arbutin and a series of parallel lines were on a Lineweaver–Burk plot, which presumed that α-arbutin was an uncompetitive activator of diphenolase. *K*_m_ and *V*_max_ values increased with increasing concentrations of β-arbutin, and the intersection of a series of lines occurred in the first quadrant. Activation type of β-arbutin was not clear. *K*_m_ values increased with increasing concentrations of deoxyarbutin, but *V*_max_ remained constant. The intersection of a series of lines was on the *y*-axis. With these results, it could be presumed that deoxyarbutin was a competitive inhibitor of diphenolase (Figure 4B,C).

### 3.5. Effects of Compounds on BTYR Activity (Tyrosinase-Mediated Dopachrome Formation)

**Figure 5:**
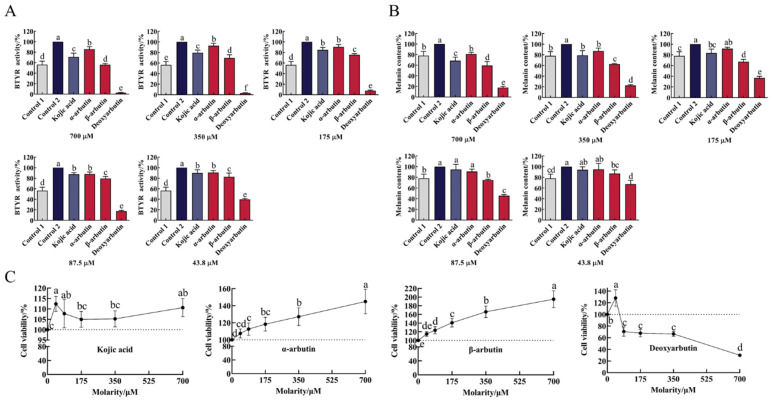
Effect of four compounds on tyrosinase activity, melanin content and cell viability of B16F10 cells. (**A**) Effect of four compounds on BTYR activity; (**B**) Effect of four compounds on melanin content; (**C**) Effect of four compounds on cell viability. Control 1 was blank group, while control 2 was control group.

As shown in Figure 5A and Table S1, B16F10 cells markedly increased tyrosinase activity upon exposure to α-MSH stimulation. At the molarity of 43.8–700 µM, all compounds significantly inhibited BTYR activity in a dose-dependent manner compared to control 2. The ability of deoxyarbutin (3 ± 1% to 40 ± 2% BTYR activity) to inhibit BTYR activity was significantly stronger than other three compounds and control 1 (56 ± 7% BTYR activity) at 43.8–700 µM. At the same molarity (43.8–700 µM), the ability of β-arbutin (56 ± 2% to 83 ± 7% BTYR activity) to inhibit BTYR activity was significantly stronger than kojic acid (71 ± 7% to 90 ± 6% BTYR activity) and α-arbutin (86 ± 5% to 91 ± 4% BTYR activity). The ability of kojic acid to inhibit BTYR activity was between α-arbutin and β-arbutin at 350–700 µM with significant difference.

### 3.6. Effects of Compounds on Melanin Content of B16F10 Cells

Upon exposure to α-MSH stimulation, B16-F10 cells markedly increased melanin content (Figure 5B and Table S2). This change was consistent with the change of BTYR activity, which indicated that α-MSH increased melanin content by promoting BTYR activity. At molarities of 43.8–700 µM, all compounds reduce melanin content in a dose-dependent manner. Compared to control 2, deoxyarbutin (43.8–700 µM), β-arbutin (43.8–700 µM), kojic acid (175–700 µM), and α-arbutin (350–700 µM) significantly reduced melanin content. The ability of deoxyarbutin (18 ± 2% to 67 ± 7% melanin content) to reduce melanin content was significantly stronger than other three compounds, β-arbutin (59 ± 8% to 87 ± 7% melanin content), kojic acid (69 ± 7% to 94 ± 5% melanin content), and α-arbutin (81 ± 3% to 95 ± 11% melanin content), at experimental molarity. It was also stronger than control 1 (78 ± 8% melanin content), which meant that 43.8–700 µM deoxyarbutin reduced melanin content less than the unstimulated level. At the same molarity (87.5–700 µM), the ability of β-arbutin to reduce melanin content was significantly stronger than kojic acid and α-arbutin. The ability of kojic acid to reduce melanin content was between α-arbutin and β-arbutin at 175–700 µM, while it was significantly stronger than α-arbutin at 700 µM.

### 3.7. Effects of Compounds on Cell Viability of B16F10 Cells

In order to verify the safety profile of this class of compounds, the cytotoxicity of kojic acid, α-arbutin, β-arbutin, and deoxyarbutin against B16F10 cells were tested by using CCK-8 assay. As shown in Figure 5C and Table S3, deoxyarbutin inhibited cell viability in a dose-dependent manner. Conversely, α-arbutin and β-arbutin promoted cell viability in a dose-dependent manner. Deoxyarbutin exhibited high cytotoxicity against B16F10 at 87.5–700 μM (71 ± 8% to 30 ± 1% cell viability). The ability of β-arbutin (115 ± 5% to 195 ± 20% cell viability) to increase cell viability was stronger than α-arbutin (108 ± 5% to 145 ± 14% cell viability) at the same molarity. Kojic acid did not affect cell viability at the concentration of 43.8–700 µM (105 ± 4% to 112 ± 4%).

## 4. Discussion

Tyrosinase, containing two copper ions located in the active site, is widely distributed in plants, animals, and microorganisms [25]. It plays an important role in melanin biosynthesis and enzymatic browning of fresh-cut fruits and vegetables [24,26]. Tyrosinase inhibitors have been usually recognized as skin-whitening agents and food preservatives [8,27]. Kojic acid and β-arbutin are well-known tyrosinase inhibitors [8,28]. They explicitly displayed the ability to inhibit browning of fruits and vegetables and reduce the prominence of stains in previous reports. Shah et al. reported that litchi fruit treated with 6 mmol/L kojic acid in pre-storage can delay pericarp browning [29]. In organ culture experiments, β-arbutin eliminated the hyperpigmentation effects of α-MSH in brownish guinea pig and human skin explants [30]. α-arbutin and deoxyarbutin, synthetic counterpart and derivative of β-arbutin, have attracted more and more attention with the in-depth study of β-arbutin. Part of the reason is that they show stronger antimelanogenic and tyrosinase inhibitory activity than β-arbutin in some studies. α-arbutin could inhibit 60% of melanin synthesis in a three-dimensional human skin model [31]. Deoxyarbutin demonstrated a rapid and sustained skin lightening effect, whereas kojic acid and β-arbutin exhibited a non-significant skin lightening effect in a hairless and pigmented guinea pig model [11].

Tyrosinase catalyzes the first two key steps in melanin biosynthesis—the hydroxylation of L-tyrosine to L-DOPA, and the oxidation of L-DOPA to dopaquinone [13]—due to possessing both monophenolase activity and diphenolase activity. Some studies reported that the same compound showed different activities on monophenolase and diphenolase. Qin et al. reported that α-arbutin inhibited monophenolase activity, whereas it activated diphenolase activity of MTYR [7]. Xu et al. [32] reported that 200 µM β-arbutin inhibited monophenolase activity and did not inhibit diphenolase activity of MTYR. Our results indicated that α-arbutin and β-arbutin had dual effect on MTYR, inhibiting monophenolase activity, but activating diphenolase activity, which is consistent with the study reported above. There are also some studies that contradict to the results of our study. Funayama et al. [23] reported that α-arbutin below 10 mM did not inhibit the diphenolase activity of MTYR, but β-arbutin could inhibit with the IC_50_ of 8.4 mM. In their experiment, 600 units of MTYR and 0.83 mM L-DOPA were used (L-DOPA/MTYR = 0.83 mM/600 U). In our study, 40 µL of 30 U/mL MTYR and 40 µL of 8 mM L-DOPA was added in 200 µL reaction system (L-DOPA/MTYR = 800 mM/600 U). The amount of substrate, L-DOPA, was sufficient in our experiment and far outweighed that in Funayama et al.’s experiment, which may be the reason why β-arbutin presented different activities on diphenolase activity of MTYR. Kojic acid could inhibit both monophenolase and diphenolase activity of MTYR. Additionally, deoxyarbutin inhibited diphenolase activity in a dose-dependent manner, while it could not inhibit monophenolase activity but only extend the lag time. In a hairless and pigmented guinea pig model, deoxyarbutin demonstrated rapid and sustained skin lightening that was completely reversible within 8 weeks after halting topical application [11]. Another report indicated that, after treating human melanocytes with deoxyarbutin for 5 days and halting the treatment for 8 days, the tyrosinase activity and melanin of human melanocytes returned to the same level as before treatment, indicating that the inhibitory effect of deoxyarbutin on tyrosinase activity is reversible [19]. These reversible phenomena of deoxyarbutin applied to skin or cells may be partly explained by its activity on monophenolase (extending lag time but not inhibiting tyrosinase activity), due to L-DOPA being almost absent in skin cells [33].

Kinetic analysis on monophenolase and diphenolase further revealed the activity mechanism of kojic acid, α-arbutin, β-arbutin, and deoxyarbutin. Garcia-Jimenez et al. demonstrated that both α- and β-arbutin were competitive inhibitors against the monophenolase activities of MTYR [6]. In another study, β-arbutin was reported to be an inhibitor in a competitive relationship with L-tyrosine [21]. In our study, α-arbutin was competitive inhibitor of monophenolase and β-arbutin was also a competitive inhibitor of monophenolase when at moderate molarity (250–1000 µM). These results were consistent with the above reports. A competitive inhibitor can bind to a free enzyme and prevent substrate from binding to the enzyme active site [4,34]. However, the inhibition type of β-arbutin seems to be influenced by its concentration. When at high molarity (4000 µM), β-arbutin was found to be a noncompetitive inhibitor of monophenolase in this study. Noncompetitive inhibitors bind to free enzymes or enzyme–substrate complexes with the same equilibrium constant [35]. Both α-arbutin and β-arbutin were activators of diphenolase of MTYR, but their inhibition types were different. Although *V*_max_ and *K*_m_ were increasing with the increased molarity of α-arbutin and β-arbutin, a series of lines with different slopes showed different intersection in Lineweaver–Burk plots of α-arbutin and β-arbutin. There were a series of parallel lines in the Lineweaver–Burk plot of α-arbutin, while there were a series of lines that intersected in first quadrant in the Lineweaver–Burk plot of β-arbutin. Therefore, α-arbutin was assumed to be an uncompetitive activator of diphenolase. The inhibition type of β-arbutin on diphenolase was not clear. Qin L. et al. [7] reported that α-arbutin acted as an activator for diphenolase activity, and its activation mechanism was a mixed-type activation. The difference of activation mechanism between uncompetitive activation in our test and mixed type activation in the research of Qin L. et al. [7] was unclear so far. Deoxyarbutin was a competitive inhibitor of diphenolase. Interestingly, kojic acid could inhibit monophenolase and diphenolase activity of MTYR, but the inhibition type of kojic acid on monophenolase and diphenolase was different. Kojic acid was a competitive inhibitor of monophenolase, while it was a mixed-type of both competitive and noncompetitive inhibitor of diphenolase, which was consistent with the results of He et al. on diphenolase activity [24].

In cellular experiment, deoxyarbutin can effectively inhibit BTYR activity and reduce melanin content. However, deoxyarbutin also potently decreased cell viability. Therefore, the ability of deoxyarbutin to inhibit BTYR activity and to reduce melanin content may be achieved by its powerful cytotoxicity. The cell viability (128.2 ± 14.02%) of deoxyarbutin at 43.8 µM was more than 90%, and is non-toxic to cells at this molarity. The melanin content and BTYR activity at 43.8 µM were 67 ± 7% and 40 ± 2%, respectively. Therefore, the concentration of deoxyarbutin that can effectively inhibit BTYR activity and melanin synthesis in cell experiments should be less than or equal to 43.8 µM, that could maintain more than 90% cell viability. Effective tyrosinase inhibitors were commonly screened as inhibiting tyrosinase activity or reducing melanin content without influencing cell viability. However, α-arbutin and β-arbutin not only dose-dependently inhibited BTYR activity and reduced melanin content, but also dose-dependently increased cell viability in this study, which was more prominent in β-arbutin. The antimelanogenic mechanism of α-arbutin and β-arbutin in cells was reported to directly inhibit catalytic activity of expressed tyrosinase without affecting the mRNA expression and protein levels of tyrosinase. Therefore α-arbutin and β-arbutin could hinder the synthesis of L-DOPA and dopaquinone, thus inhibiting the production of melanin [20,31,32,36]. β-arbutin did not change the molecular size or the content of tyrosinase, DHICA oxidase (TRP1), or dopachrome tautomerase 2 (TRP2), but it might inhibit the melanogenesis process of the rest of the enzymes at the post-translational level [36]. As Akiu et al. [20] reported that β-arbutin reduced the melanin content of cultured murine melanoma B16 cells by decreasing intracellular tyrosinase activity. Consequently, it can be assumed that the increase in cell viability is to spontaneously reduce the BTYR catalytic activity in cells and then reduce the content of melanin. A study reported that 0.25–0.5 mM α-arbutin reduced melanin content and decreasing tyrosinase activity in a dose dependent manner, but 0.25–1 mM α-arbutin did not influence cell proliferation in cultured human melanoma cells [31]. The activity difference with our result on cell viability may result in the difference of origins of the tyrosinase (cultured human melanoma cells/B16F10 mouse malignant melanoma cells) and cultured time with α-arbutin (6 d/2 d). Another study reported that the cytotoxicity of tert-butyl hydroperoxid (t-BHP) was significantly reduced by β-arbutin pretreatment in Hep-G2 cell line [37], which indicated that β-arbutin has the ability to protect cells and improve cell viability in some cases. However, the ability of α-arbutin and β-arbutin to improve cell viability of B16F10 cells in a dose-dependent manner is worthy of close attention. B16F10 cell is a kind of tumor cell. It is a very important consideration index and a very serious side effect when applied to human skin care products or fruit and vegetable preservatives if α-arbutin and β-arbutin can indeed promote the viability of tumor cells. Therefore, this phenomenon needs to be further verified in other cells. Kojic acid did not affect cell viability at 43.8–700 µM, but inhibited BTYR activity and reduced melanin content in a dose-dependent manner. It showed that kojic acid reduced melanin content, due to inhibiting BTYR activity rather than possessing cytotoxicity.

Inhibitory activity of kojic acid, α-arbutin, β-arbutin, and deoxyarbutin on tyrosinase was compared. In the ability of inhibiting monophenolase activity of MTYR, kojic acid was stronger than β-arbutin, and β-arbutin was stronger than α-arbutin. All of them extended the lag time and reduced the enzyme activity in the steady state. Deoxyarbutin only extended the lag time. Kiato et al. reported that the ability of α-arbutin to inhibit monophenolase activity of MTYR was weaker than β-arbutin [14]. The IC_50_ of α-arbutin and β-arbutin on the inhibitory activity of MTYR monophenolase was 8 mM and 0.9 mM respectively [6], which meant β-arbutin possessing stronger inhibitory activity than α-arbutin. These results were consistent with our result. As for the ability of inhibiting diphenolase activity of MTYR, kojic acid was stronger than deoxyarbutin. Diphenolase promoting activity of α-arbutin at 4000–8000 µM was stronger than β-arbutin at the same molarity. That means α-arbutin and β-arbutin both act as activators in the reaction of catalyzing L-DOPA into dopachrome by diphenolase. Different from the above results about diphenolase activity of MTYR, all compounds inhibit intracellular BTYR activity (diphenolase activity) in a dose-dependent manner at the molarity of 43.8–700 µM. At 43.8 µM, the cell viabilities of all compounds were among 108 ± 5% to 128 ± 14%, that is cytotoxicity of deoxyarbutin and activation of α-arbutin and β-arbutin on B16F10 cells being minimized as far as possible, so the ability of four compounds inhibiting BTYR activity can be compared. All compounds (43.8 µM) could significantly inhibit diphenolase activity of BTYR. Deoxyarbutin was significantly stronger than β-arbutin and attenuated α-MSH-induced BTYR activity lower than the unstimulated states (control 1). β-arbutin was significantly stronger than kojic acid and α-arbutin. There was no significant difference between kojic acid and α-arbutin. Maeda et al. [38] showed that β-arbutin inhibited tyrosinase activity in human melanocytes in a dose dependent manner (0.1 to 1.0 mM) without significantly decreasing cell viability, and it reduced cellular melanin synthesis more potent than kojic acid when compared at 0.5 mM. This stronger activity to reduce melanin content of β-arbutin compared with kojic acid was consistent with our results. There are some contradictory results in published papers. Funayama et al. reported that the inhibitory activity of α-arbutin on tyrosinase from B16 mouse melanoma was 10 times that of β-arbutin [23]. Sugimoto et al. reported that α-arbutin is more potent than β-arbutin as an inhibitor of the tyrosinase (L-DOPA as the substrate) from human malignant melanoma cells, HMV-II [13]. These results obtained by using the tyrosinase from mammalian cells indicated that a-arbutin is a more effective skin-whitening agent than β-arbutin. It is worth noting that, in the assay of tyrosinase activity reported by Funayama [23] and Sugimoto [13], tyrosinase in the mammalian cell was extracted first, and then reacted with substrate (L-DOPA) in the presence of samples. While in our experiment, B16F10 cells was cultured by medium containing samples first and intracellular tyrosinase activity was influenced by samples in this procedure. Then, tyrosinase in the cells was extracted, followed by reacting with L-DOPA. The effect of α-arbutin and β-arbutin on tyrosinase (diphenolase) may be affected by the method in which α-arbutin and β-arbutin come into contact with the tyrosinase. It can be presumed that α-arbutin was stronger than β-arbutin in direct contact, while β-arbutin was stronger than α-arbutin in indirect contact with mammalian tyrosinase (diphenolase). Additionally, the different law of inhibitory activity in cell system and cell free system may be influenced by the origin of tyrosinases. Since MTYR is commercially available, it might have been thought to be useful for the first screening of a tyrosinase inhibitor. However, the amino acid sequence identity between human tyrosinase and MTYR (Gene bank accession no. O42713) is only 23%. Therefore, the tyrosinase from mushroom could not always be used as a model enzyme to obtain the inhibitor of tyrosinase from mammalian origins [39]. On the other hand, human tyrosinase and murine tyrosinase are highly homologous with 82% sequence identity. Therefore, it is definitely important to use human tyrosinase for screening skin-whitening agents [13].

This study did not explain all contradictory results that have been reported before. However, the activities of four compounds in mushroom and mouse cells tyrosinase were studied as much as possible under unified experimental conditions, such as the origin and purity of the enzyme, the conformational state of the enzyme, the type and concentration of substrates, oxygen concentration, pH, temperature, and the purity of tested compounds.

## 5. Couclusions

Based on the results of mushroom monophenolase and diphenolase activity experiment and cell experiment, kojic acid was considered the most suitable positive control among the four compounds because it could inhibit monophenolase and diphenolase activity of MTYR and reduce intercellular melanin content by inhibiting BTYR activity without cytotoxicity. Activity difference and inhibition type of four compounds on monophenolase and diphenolase of MTYR were exposed. Their effects on intercellular BTYR activity, melanin content, and cell viability were also systematically studied. Some interesting results were found. Firstly, both α-arbutin and β-arbutin had a dual effect on mushroom tyrosinase, inhibiting monophenolase activity but activating diphenolase activity. Secondly, both α-arbutin and β-arbutin promote cell viability in a dose-dependent manner, and β-arbutin was stronger than α-arbutin. Thirdly, deoxyarbutin did not reduce the activity but it increased the lag time of monophenolase of mushroom tyrosinase. Fourthly, diphenolase inhibitory activity difference in cell-free and cell systems, may be a result of the different origins of the tyrosinases, mushroom tyrosinase and murine tyrosinase. In order to explain some novel phenomena found in this study, a mechanism should be developed further, such as changes in gene and protein expression in pathways related to melanin synthesis and cell viability. Melanin transfer and release mechanisms may also be influencing factors. Moreover, considering the difference of tested compounds on monophenolase and diphenolase activity of MTYR, the difference of tested compounds on monophenolase and diphenolase inhibition type of MTYR, the difference of tested compounds on diphenolase activity of MTYR and BTYR, clinical experiments (animal or human skins), and browning experiments of fruits and vegetables could provide more valuable and useful results compared with only using MTYR as test material.

## Data Availability

The data are contained within the article and Supplementary Material.

## Supplementary Materials

The following supporting information can be downloaded at: https://www.mdpi.com/article/10.3390/antiox11030502/s1, Table S1: Effect of compounds on tyrosinase activity of B16F10 cells stimulated by α-MSH; Table S2: Effect of compounds on melanin content of B16F10 cells stimulated by α-MSH; Table S3: Effect of compounds on cell viability of B16F10 cells.

## References

[1] Wang W., Chen L., Wang W.W., Zhang J., Jiang H. (2021). Inhibition of active compounds in tea on melanin formation. J. Tea Sci..

[2] Hu Y.H., Chen Q.X., Cui Y., Gao H.J., Xu L., Yu X.Y., Wang Y., Yan C.L., Wang Q. (2016). 4-Hydroxy cinnamic acid as mushroom preservation: Anti-tyrosinase activity kinetics and application. Int. J. Biol. Macromol..

[3] Moon K.M., Lee B., Cho W.K., Lee B.S., Kim C.Y., Ma J.Y. (2018). Swertiajaponin as an anti-browning and antioxidant flavonoid. Food Chem..

[4] Zolghadri S., Bahrami A., Khan M.T.H., Munoz-Munoz J., Garcia-Molina F., Garcia-Canovas F., Saboury A.A. (2019). A comprehensive review on tyrosinase inhibitors. J. Enzym. Inhib. Med. Chem..

[5] Lee H.K., Ha J.W., Hwang Y.J., Boo Y.C. (2021). Identification of L-cysteinamide as a potent inhibitor of tyrosinase-mediated dopachrome formation and eumelanin synthesis. Antioxidants.

[6] Garcia-Jimenez A., Teruel-Puche J.A., Berna J., Rodriguez-Lopez J.N., Tudela J., Garcia-Canovas F. (2017). Action of tyrosinase on alpha and beta-arbutin: A kinetic study. PLoS ONE.

[7] Qin L., Wu Y., Liu Y., Chen Y., Zhang P. (2014). Dual Effects of alpha-arbutin on monophenolase and diphenolase activities of mushroom tyrosinase. PLoS ONE.

[8] He M., Fan M., Peng Z., Wang G. (2021). An overview of hydroxypyranone and hydroxypyridinone as privileged scaffolds for novel drug discovery. Eur. J. Med. Chem..

[9] Shao L.L., Wang X.L., Chen K., Dong X.W., Kong L.M., Zhao D.Y., Hider R.C., Zhou T. (2018). Novel hydroxypyridinone derivatives containing an oxime ether moiety: Synthesis, inhibition on mushroom tyrosinase and application in anti-browning of fresh-cut apples. Food Chem..

[10] Wang W., Chen L., Wang W.W., Zhang J.Y., Engelhardt U.H., Jiang H.Y. (2022). Effect of active groups and oxidative dimerization on the antimelanogenic activity of catechins and their dimeric oxidation products. J. Agric. Food Chem..

[11] Boissy R.E., Visscher M., DeLong M.A. (2005). Deoxyarbutin: A novel reversible tyrosinase inhibitor with effective in vivo skin lightening potency. Exp. Dermatol..

[12] Anwar A.I., Asmarani Y., Madjid A., Patellongi I., Adriani A., As’ad S., Kurniadi I. (2021). Comparison of 2% deoxyarbutin and 4% hydroquinone as a depigmenting agent in healthy individuals: A double-blind randomized controlled clinical trial. J. Cosmet. Dermatol..

[13] Sugimoto K., Nishimura T., Nomura K., Sugimoto K., Kuriki T. (2003). Syntheses of arbutin-alpha-glycosides and a comparison of their inhibitory effects with those of alpha-arbutin and arbutin on human tyrosinase. Chem. Pharm. Bull..

[14] Kitao S., Sekine H. (1994). α-D-Glucosyl transfer to phenolic compounds by sucrose phosphorylase from leuconostoc mesenteroides and production of α-arbutin. Biosci. Biotechnol. Biochem..

[15] Liu C.Q., Deng L., Zhang P., Zhang S.R., Liu L., Xu T., Wang F., Tan T.W. (2013). Screening of high α-arbutin producing strains and production of α-arbutin by fermentation. World J. Microbiol. Biotechnol..

[16] Seo D.H., Jung J.H., Lee J.E., Jeon E.J., Kim W., Park C.S. (2012). Biotechnological production of arbutins (α- and β-arbutins), skin-lightening agents, and their derivatives. Appl. Microbiol. Biotechnol..

[17] Xu W.H., Liang Q., Zhang Y.J., Zhao P. (2015). Naturally occurring arbutin derivatives and their bioactivities. Chem. Biodivers..

[18] Saeedi M., Khezri K., Zakaryaei A.S., Mohammadamini H. (2021). A comprehensive review of the therapeutic potential of α-arbutin. Phytother. Res..

[19] Chawla S., DeLong M.A., Visscher M.O., Wickett R.R., Manga P., Boissy R.E. (2008). Mechanism of tyrosinase inhibition by deoxyarbutin and its second-generation derivatives. Br. J. Dermatol..

[20] Akiu S., Suzuki Y., Asahara T., Fujinuma Y., Fukuda M. (1991). Inhibitory effect of arbutin on melanogenesis—Biochemical study using cultured B16 melanoma cells. Nihon Hifuka Gakkai Zasshi.

[21] Maeda K., Fukuda M. (1996). Arbutin: Mechanism of its depigmenting action in human melanocyte culture. J. Pharmacol. Exp. Ther..

[22] Nakajima M., Shinoda I., Fukuwatari Y., Hayasawa H. (1998). Arbutin increases the pigmentation of cultured human melanocytes through mechanisms other than the induction of tyrosinase activity. Pigment Cell Res..

[23] Funayama M., Arakawa H., Yamamoto R., Nishino T., Shin T., Murao S. (1995). Effects of α- and β-arbutin on activity of tyrosinases from mushroom and mouse melanoma. Biosci. Biotechnol. Biochem..

[24] He M., Fan M., Liu W., Li Y., Wang G. (2021). Design, synthesis, molecular modeling, and biological evaluation of novel kojic acid derivatives containing bioactive heterocycle moiety as inhibitors of tyrosinase and antibrowning agents. Food Chem..

[25] Matoba Y., Kumagai T., Yamamoto A., Yoshitsu H., Sugiyama M. (2006). Crystallographic evidence that the dinuclear copper center of tyrosinase is flexible during catalysis. J. Biol. Chem..

[26] Korner A., Pawelek J. (1982). Mammalian tyrosinase catalyzes three reactions in the biosynthesis of melanin. Science.

[27] Carcelli M., Rogolino D., Bartoli J., Pala N., Compari C., Ronda N., Bacciottini F., Incerti M., Fisicaro E. (2020). Hydroxyphenyl thiosemicarbazones as inhibitors of mushroom tyrosinase and antibrowning agents. Food Chem..

[28] Ghofrani N.S., Sheikhi M., Amirzakaria J.Z., Hassani S., Haghbeen K. (2021). New insight into the interactions of arbutin with mushroom tyrosinase. Protein J..

[29] Shah H.M.S., Khan A.S., Ali S. (2017). Pre-storage kojic acid application delays pericarp browning and maintains antioxidant activities of litchi fruit. Postharvest Biol. Technol..

[30] Lim Y.J., Lee E.H., Tong H.K., Sang K.H., Oh M.S., Kim S.M., Yoon T.J., Kang C., Park J.H., Sun Y.K. (2009). Inhibitory effects of arbutin on melanin biosynthesis of α-melanocyte stimulating hormone-induced hyperpigmentation in cultured brownish guinea pig skin tissues. Arch. Pharmacal Res..

[31] Sugimoto K., Nishimura T., Nomura K., Sugimoto K., Kuriki T. (2004). Inhibitory effects of alpha-arbutin on melanin synthesis in cultured human melanoma cells and a three-dimensional human skin model. Biol. Pharm. Bull..

[32] Xu H., Li X., Xin X., Mo L., Zou Y., Zhao G., Yu Y., Chen K. (2021). Antityrosinase mechanism and antimelanogenic effect of arbutin esters synthesis catalyzed by whole-cell biocatalyst. J. Agric. Food Chem..

[33] Liu Y.T. (2021). Study on the Whitening Mechanism, Compatibility and Stability of α-Arbutin. Master’s Thesis.

[34] Fernandes M.S., Kerkar S. (2017). Microorganisms as a source of tyrosinase inhibitors: A review. Ann. Microbiol..

[35] Zhao D.Y., Zhang M.X., Dong X.W., Hu Y.Z., Dai X.Y., Wei X., Hider R.C., Zhang J.C., Zhou T. (2016). Design and synthesis of novel hydroxypyridinone derivatives as potential tyrosinase inhibitors. Bioorg. Med. Chem. Lett..

[36] Chakraborty A.K., Funasaka Y., Komoto M., Ichihashi M. (1998). Effect of arbutin on melanogenic proteins in human melanocytes. Pigment Cell Melanoma Res..

[37] Seyfizadeh N., Mahjoub S., Zabihi E., Moghadamnia A., Pouramir M., Mir H., Khosravifarsani M., Elahimanesh F. (2012). Cytoprotective effects of arbutin against tert-butyl hydroperoxid induced toxicity in Hep-G2 cell line. World Appl. Sci. J..

[38] Maeda K., Fukuda M. (1991). In vitro effectiveness of several whitening cosmetic components in human melanocytes. J. Soc. Cosmet. Chem..

[39] Funayama M., Nishino T., Hirota A., Murao S., Takenishi S., Nakano H. (1993). Enzymatic synthesis of (+)catechin-α-glucoside and its effect on tyrosinase activity. Biosci. Biotechnol. Biochem..

