# Supplementary material for: Kojic Acid Showed Consistent Inhibitory Activity on Tyrosinase from Mushroom and in Cultured B16F10 Cells Compared with Arbutins
Source: Antioxidants (Basel). 2022 Mar 4;11(3):502. doi: 10.3390/antiox11030502 (PMC8944748; doi:10.3390/antiox11030502)
Supplement: Supplementary file 1 [file antioxidants-11-00502-s001.zip › antioxidants-1615718-supplementary.pdf]

Supplementary Material

Table S1. Effect of compounds on tyrosinase activity of B16F10 cells stimulated by α-MSH.

Compounds	Concentration (μM)				
	700	350	175	87.5	43.8
Kojic acid	71 ± 7	79 ± 5	85 ± 5	88 ± 3	90 ± 6
α-arbutin	86 ± 5	93 ± 5	90 ± 5	88 ± 4	91 ± 4
β-arbutin	56 ± 2	70 ± 6	76 ± 2	79 ± 4	83 ± 7
deoxyarbutin	3 ± 1	3 ± 1	8 ± 1	17 ± 2	40 ± 2

Note: The tyrosinase activity was calculated as a percentage of the control 2. Tyrosinase activity of blank group (control 1) and control group (control 2) was 56 ± 7% and 100%, respectively.

Table S2. Effect of compounds on melanin content of B16F10 cells stimulated by α-MSH.

Compounds	Concentration (μM)				
	700	350	175	87.5	43.8
Kojic acid	69 ± 7	79 ± 9	84 ± 7	95 ± 10	94 ± 5
α-arbutin	81 ± 3	87 ± 5	92 ± 3	91 ± 5	95 ± 11
β-arbutin	59 ± 8	63 ± 1	67 ± 5	75 ± 1	87 ± 7
deoxyarbutin	18 ± 2	23 ± 2	37 ± 3	46 ± 2	67 ± 7

Note: The melanin content was calculated as a percentage of the control 2. Melanin content of control 1 and control 2 was 78 ± 8% and 100%, respectively.

Table S3. Effect of compounds on cell viability of B16F10 cells.

Compounds	Concentration (μM)				
	700	350	175	87.5	43.8
Kojic acid	111 ± 4	105 ± 4	105 ± 4	108 ± 7	112 ± 4
α-arbutin	145 ± 14	127 ± 10	118 ± 8	113 ± 7	108 ± 5
β-arbutin	195 ± 20	166 ± 13	141 ± 10	124 ± 8	115 ± 5
deoxyarbutin	30 ± 1	66 ± 4	68 ± 6	71 ± 8	128 ± 14

Note: The cell viability was calculated as a percentage of the control (no compound treated group).
